# Immunomodulatory and anti-oxidative effect of the direct TRPV1 receptor agonist capsaicin on Schwann cells

**DOI:** 10.1186/s12974-020-01821-5

**Published:** 2020-05-06

**Authors:** Thomas Grüter, Alina Blusch, Jeremias Motte, Melissa Sgodzai, Hussein Bachir, Rafael Klimas, Björn Ambrosius, Ralf Gold, Gisa Ellrichmann, Kalliopi Pitarokoili

**Affiliations:** Department of Neurology, St. Josef-Hospital, Ruhr University Bochum, Gudrundstr. 56, 44791 Bochum, Germany

**Keywords:** Capsaicin, Schwann cell culture, Chronic inflammatory demyelinating polyneuropathy, Inflammation, Oxidative stress

## Abstract

**Background:**

Only few studies describe the impact of nutritive factors on chronic inflammatory demyelinating polyneuropathy (CIDP), an inflammatory disease of the peripheral nervous system. The active component of chili pepper, capsaicin, is the direct agonist of the transient receptor potential channel vanilloid subfamily member 1. Its anti-inflammatory effect in the animal model experimental autoimmune neuritis (EAN) has been previously demonstrated.

**Methods:**

In the present study, we describe the anti-inflammatory and anti-oxidative influence of capsaicin on Schwann cells (SCs) in an in vitro setting. Hereby, we analyze the effect of capsaicin on Schwann cells’ gene expression pattern, major histocompatibility complex class II (MHC-II) presentation, and H_2_O_2_-induced oxidative stress. Furthermore, the effect of capsaicin on myelination was examined in a SC-dorsal root ganglia (DRG) coculture by myelin basic protein staining. Finally, in order to investigate the isolated effect of capsaicin on SCs in EAN pathology, we transplant naïve and capsaicin pre-treated SCs intrathecally in EAN immunized rats and analyzed clinical presentation, electrophysiological parameters, and cytokine expression in the sciatic nerve.

**Results:**

In SC monoculture, incubation with capsaicin significantly reduces interferon gamma-induced MHC-II production as well as toll-like receptor 4 and intercellular adhesion molecule 1 mRNA expression. Calcitonin gene-related peptide mRNA production is significantly upregulated after capsaicin treatment. Capsaicin reduces H_2_O_2_-induced oxidative stress in SC in a preventive, but not therapeutic setting. In a SC-DRG coculture, capsaicin does not affect myelination rate. After intrathecal transplantation of naïve and capsaicin pre-treated SCs in EAN-immunized rats, naïve, but not capsaicin pre-treated intrathecal SCs, ameliorated EAN pathology in rats.

**Conclusions:**

In conclusion, we were able to demonstrate a direct immunomodulatory and anti-oxidative effect of capsaicin in a SC culture by reduced antigen presentation and expression of an anti-inflammatory profile. Furthermore, capsaicin increases the resistance of SCs against oxidative stress. A primary effect of capsaicin on myelination was not proven. These results are in concordance with previous data showing an anti-inflammatory effect of capsaicin, which might be highly relevant for CIDP patients.

## Introduction

Acute inflammatory demyelinating polyneuropathy (AIDP)/Guillain-Barré syndrome (GBS) and chronic inflammatory demyelinating polyneuropathy (CIDP) are inflammatory diseases of the peripheral nervous system (PNS). The prevalence of CIDP ranges from 0.8 to 8.9 cases of 100,000 population depending on geographical region. However, relevant environmental factors have not been adequately investigated [1–3]. Clinical features of GBS and CIDP are tetraparesis, sensory deficits, and areflexia. Despite immune treatment with intravenous immunoglobulins, corticosteroids, and plasma exchange, more than 25% of CIDP patients remain disabled with a severe impairment of sensorimotor function [4]. Pathophysiologically, GBS and CIDP are characterized by a dysregulated autoimmune response resulting among others in an inflammation of the peripheral nerves, oxidative stress, Schwann cell (SC) apoptosis, and demyelination [5].

SCs have a dual function in the peripheral nervous system: they enable saltatory conduction, but also serve immunomodulatory and nutritive functions. Thereby, SCs prevent oxidative stress and modulate immune responses by cytokine production and antigen presentation [6]. Beyond that, recent studies have shown axonal nerve regeneration and repair after nerve injury via SC reprogramming [7]. In the case of CIDP, the interaction of SCs and neurons is disrupted, which might interfere with peripheral inflammation or re-myelination [8, 9].

The impact of environmental and nutritive factors on inflammatory diseases of the central nervous system has been extensively studied in recent years. For multiple sclerosis, the influence of vitamin D [10], the Epstein Barr virus [11], and short- and long-chain fatty acids [12] as well as combined sodium chloride and saturated long-chain fatty acids [13] have been intensively discussed. For the PNS, available data is rare. Even though worldwide studies are lacking, there is a trend to a higher GBS incidence in Europe and North America in comparison to China, Hong Kong, and Brazil [1], countries with high consumption of spicy food. Moreover, spicy diet is inversely related with total and specific mortality [14, 15].

Capsaicin is the active component of chili pepper and acts as a direct agonist of the transient receptor potential channel vanilloid subfamily member 1 (TRPV1). The expression of TRPV1 is described in the immune system as well as in the nervous system [16–20]. In previous studies, our group was able to illustrate an anti-inflammatory effect of capsaicin in the experimental autoimmune neuritis (EAN), the classical animal model of immune-mediated neuropathies. Lewis rats treated orally with capsaicin 10 days prior to EAN induction showed significant ameliorated paraparesis, improved electrophysiological function, and reduced inflammation of the sciatic nerve in histochemistry and at cytokine level. An increase in TRPV1 receptor expression and a tendency for an increased expression of its downstream molecule calcitonin gene-related peptide (CGRP) in the sciatic nerve indicated a possible receptor-dependent underlying mechanism, although it remains unclear which cell type exerts this effect [21].

In the present study, we describe the relevance of SCs for the previously described anti-inflammatory in vivo effect of capsaicin in EAN by analyzing SCs in an in vitro model. Hereby, we examine a possible anti-oxidative and immunomodulatory effect of SCs in a SC-monoculture as well as a potential beneficial effect of capsaicin on myelination in a SC-dorsal root ganglia (DRG) coculture. Finally, in order to investigate the isolated effect of capsaicin on SCs in EAN pathology, we transplanted naïve and capsaicin pre-treated SCs intrathecally in EAN immunized rats.

Here, we were able to demonstrate for the first time that capsaicin mediates an anti-oxidative and immunomodulatory effect on SCs, but does not exert a further direct influence on myelination.

## Material and methods

### Animal experiments

The present study was carried out in accordance with the European Communities Council Directive of September 22nd 2010 (2010/63/EEC) for care of laboratory animals and after approval of the local government committee for nature, environment, and consumer protection (TVA 81-02.04.2017.A482). Male Sprague-Dawley rats (for SC extraction, five animals per SC culture experiment, 3–4 weeks old, Charles River, Erkrath, Germany), pregnant Sprague-Dawley rats (for DRG extraction, one animal per coculture experiment, E14, Charles River), and female Lewis rats (for EAN induction, 7–8 weeks old, Charles River) were housed in a temperature- and humidity-controlled vivarium with a constant 12-h light/dark cycle (lights on from 6 a.m.to 6 p.m.) with *ad libitum* food and water access. All surgical procedures and experiments were conducted during the day.

### Capsaicin

Capsaicin (Alps Pharmaceutical, Hida, Japan, 93.1% pure powder) was dissolved in dimethyl sulfoxide (DMSO). Beside coculture experiments, capsaicin was used in a concentration of 10 μM and a corresponding DMSO concentration of 0.1%.

### Assessment of the oxidative and modulatory potential of Schwann cells

#### Schwann cell culture

SC preparation was conducted as previously described [22] based on the protocol of Andersen and colleagues [23]. After sacrificing rats by decapitation, sciatic nerves were sampled in sterile phosphate-buffered saline (PBS) and transferred in Leibovitz’s L-15 medium enriched with 50 μg/ml Gentamycin (Thermo Fisher Scientific, Waltham, MA, USA). Nerves were stripped of epineurium and sectioned into 1–2 mm pieces. Explants were dissociated for 18 h (37 °C, 5% CO_2_) with 1.25 U/ml dispase II (0.25%) (Merck, Darmstadt, Germany) and 0.05% type I collagenase (Merck) in 580 mg/l l-glutamine and 4500 mg/l glucose-enriched Dulbecco’s modified Eagle’s medium (DMEM, Thermo Fisher Scientific) with 50 μg/ml Gentamycin. After stopping dissociation with Hank’s balanced salt solution (HBSS, Thermo Fisher Scientific) containing 40% fetal bovine serum (Merck), suspension was filtered through a 100-μm strainer. SC cultures were placed overnight on poly-l-lysine (Merck) and 1 μg/cm^2^ laminin (Merck) coated dishes in DMEM containing 10% fetal bovine serum and 50 μg/ml Gentamycin. Supplementation of the culture medium with 10 nM neuregulin (PeproTech, Hamburg, Germany) and 2 μM forskolin (Merck) as early as 1 day after plating rapidly expanded the SC population. Low rate of fibroblast contamination was maintained by magnetic cell sorting selecting Thy-1-positive fibroblasts as described by the manufacturer’s protocol (Miltenyi Biotec, Bergisch Gladbach, Germany).

#### Flow cytometric analyses of Schwann cells

Adherent SCs were released from coated dishes with trypsin/EDTA (Merck) for 5 min at 37°. In order to analyze SC monoculture purity, we stained for SOX10 as a SC marker (1:1000, rabbit recombinant monoclonal antibody, Abcam, Cambridge, UK) after selection of viable cells (fixable viability dye eFluor 780, Thermo Fisher Scientific) as described by the manufacturer’s protocol. To exclude toxicity of capsaicin, we performed titration experiments and subsequent propidium iodide (PI) staining of SCs (Thermo Fisher Scientific) as described by the manufacturer’s protocol. Therefore, SC were incubated with capsaicin and corresponding DMSO in concentrations of a range from 0.1 μM to 1 mM over 24 h. Flow cytometry of single cell suspensions were performed with a FACS Canto II (BD Biosciences, NJ, USA) and DIVA Software (BD Pharmingen, NJ, USA)

#### Immunocytochemical staining

For immunocytochemical analyses, SCs were seeded and incubated on poly-l-lysine- and laminin-coated coverslips for 2 days. After fixation with 4% paraformaldehyde, permeabilization with 0.1% PBS-Triton, and blocking with 10% normal goat serum, the cells were exposed to the primary antibody S-100 (mouse-anti-rat, 1:500, Merck), SOX10 (rabbit-anti-rat, 1:1000, Abcam), or TRPV1 (rabbit-anti-rat, 1:1000, Thermo Fisher Scientific). Immunoreaction was detected with the secondary antibody, goat-anti-mouse IgG-Alexa Flour 488 (1:1000, Thermo Fisher Scientific), or goat-anti-rabbit IgG-Alexa Flour 488 (1:1000, Thermo Fisher Scientific), respectively. Furthermore, nuclei were counterstained with DAPI (4′,6′-diamidino-2-phenylindole·2HCl, Biozol Diagnostica Vertrieb, Eching, Germany). The omission of the primary antibodies served as negative control. Specificity of the staining was also controlled on sections of fibroblasts.

#### Schwann cell stimulation with Interferon gamma

In order to explore the effect of capsaicin on antigen presentation of SCs, we generated a sub-maximal major histocompatibility complex class II (MHC-II) surface presentation after incubation with 100 U/ml rat interferon (IFN) gamma for 5 days in accordance to previous experiments [22, 24]. To examine the MHC-II presentation after IFN gamma stimulation with capsaicin or vehicle treatment, we stained with MHC-II Alexa 647 antibody (mouse monoclonal antibody, 1:100, BD Biosciences) and analyzed with flow cytometry as described above.

#### Quantitative real-time PCR

Total RNA was isolated using the RNeasy Mini extraction kit (QIAGEN, Naamloze, Netherlands). All samples were treated with the RNA Stabilization Reagent (RNAlater, Qiagen) at 37 °C overnight and stored at − 80 °C until use. Total RNA was reverse-transcribed into cDNA as described by the manufacturer’s protocol for the Reverse Transcription System (Promega, Fitchburg, WI, USA). The following sequence-specific sense (sen) and anti-sense (ase) primers were designed and mRNA expression levels were analyzed by qrt-PCR according to the manufacturer’s instructions (Thermo Fisher Scientific): IFN gamma (sen AAA GAC AAC CAG GCC ATC AG, ase CTT TTC CGC TTC CTT AGG CT), interleukin 10 (IL-10, sen CCT GCT CTT ACT GGC TGG AG, ase TCT CCC AGG GAA TTC AAA TG), interleukin 4 (IL-4, sen TGA TGG GTC TCA GCC CCC ACC TTG C, ase CTT TCA GTG TTG TGA GCG TGG ACT C), TNF alpha (sen CCA CCA CGC TCT TCT GTC TA, ase GCC ATG GAA CTG ATG AGA GG), TRPV1 (sen CTT CTG AGG GAT GCA AGC AC, ase CCT GGG ACC ATG GAA TCC TT), calcitonin gene-related peptide (CGRP, sen CCT TCG GGT CTG AGG AAC TA, ase GGC GTG GTG AGT TCA ACT TT), intercellular adhesion molecule 1 (ICAM-1, sen ACC ACC TCC CCA CCT ACA TAC, ase ACA TTT TCT CCC AGG CAT TCT), and toll-like receptor 4 (TLR4, sen GCG CCT AAA ACC CAT TAT GTT, ase TGA TTC TTT GCC TGA GTT GCT). RT-PCR was performed by using the RT-PCR System 7500 (Thermo Fisher Scientific). Relative gene quantitation of mRNA expression was performed using the comparative CT-method and analysis was conducted according to Pfaffl [25]. In this context, the relative expression ratio is calculated only from the rt-PCR efficiencies and the crossing point deviation of the sample versus a control. Relative target mRNA expression was normalized to the geometric expression average of the housekeeping genes β-actin and glyceraldehyde 3-phosphate dehydrogenase. We applied each sample in two technical replicates. The mean Ct was used in the equation for the housekeeping genes and Ct for the genes of interest.

#### Application of oxidative stress (H_2_O_2_)

We performed titration experiments (PI staining in flow cytometry) on SCs with H_2_O_2_ concentrations from 3 μM to 3 mM for 24 h to evaluate rate of cell death by oxidative stress. Afterwards, we incubated SCs for 24 h with 30 μM H_2_O_2_ with simultaneous treatment of capsaicin or vehicle to examine the protective role of capsaicin in the context of oxidative stress. Furthermore, we repeated these experiments with further pre-treatment of 48 h with 10 μM capsaicin or vehicle to simulate a protective effect of capsaicin at a preventive setting.

### Schwann cell-dorsal root ganglion-coculture

#### Dorsal root ganglion extraction and coculture preparation

Pregnant Sprague-Dawley rats were sacrificed via CO_2_ followed by decapitation at pregnancy day 14 and the DRGs of the embryos were extracted. Single DRG explants were placed on consecutively 0.2 mg/ml poly-d-lysine (Sigma-Aldrich, St. Louis, MO, USA) and 1 μg/ml laminin (Sigma-Aldrich) coated coverslips in neurobasal medium (Invitrogen, Carlsbad, CA, USA) containing 2% B27 (Life Technologies, CA, USA), 2% normal horse serum (Thermo Fischer Scientific), 1% l-glutamine (Thermo Fischer Scientific), and 0.5% penicillin/streptomycin (Thermo Fischer Scientific). Further, 10 ng/ml neuronal growth factor was added freshly to the medium before use. After 2 days of cultivation at 37 °C and 5% CO_2_, we added 20,000 SCs per well in the abovementioned medium additionally supplemented with 50 μg/ml ascorbic acid (Sigma-Aldrich). Medium containing neuronal growth factor and ascorbic acid was used until the end of the experiment. For analysis of an increased myelination over time, coculture was conducted until day 16. We applied 0.1 μM, 1 μM, and 10 μM capsaicin to the coculture changing medium every second day. Statistical analysis between capsaicin and DMSO-treated control coculture was performed at coculture day 14.

#### Immunocytochemical evidence of myelination

To evaluate myelination in vitro, we stained the coculture for beta III tubulin and myelin basic protein (MBP). After fixation with 4% paraformaldehyde and blocking with 10% normal goat serum (Vector Laboratories, Burlingame, CA, USA) and 0.5% Triton X100 (Sigma-Aldrich) in PGBA (0.1 M phosphate buffer with 0.5% gelatin and 1% bovine serum albumin), the cells were exposed to the primary antibody beta III tubulin (rabbit monoclonal antibody, T-2200, Sigma-Aldrich) and MBP (mouse monoclonal antibody, ABIN446360, Novus Biologicals, Centennial, CO, USA) 1:7500 and 1:750 diluted in blocking solution, respectively. Immunoreaction was detected with the secondary antibodies, goat anti-mouse IgG Alexa Flour 488 (Invitrogen) and goat anti-rabbit IgG Alexa Flour 568 (Invitrogen) diluted 1:1000 in blocking solution. Nuclei were counterstained with DAPI. Afterwards, we measured the area of MBP-positive axons in relation to the area of beta III tubulin axons to obtain the myelination rate of the coculture.

### Intrathecal transplantation of capsaicin-treated Schwann cells in the experimental autoimmune neuritis animal model

#### In vivo study design

We evaluated the isolated influence of naïve and capsaicin-treated SC in the EAN model. Therefore, we injected pure PBS, SCs in PBS, or 24 h 10 μM capsaicin-treated SCs in PBS in the EAN rat 11 days after immunization. The outcome was measured by clinical scoring, electrophysiology, and qrt-PCR.

#### Experimental autoimmune neuritis

EAN induction was conducted as described earlier [21, 22]. In short, 7–8-week-old female Lewis rats were anesthetized by exposure to 1.5–2.0% halothane in oxygen. Afterwards, rats were immunized by subcutaneous injection of 250 μg neuritogenic P2_53-78_ (synthesized by Dr. Rudolf Volkmer from Charité–Universitätsmedizin Berlin, Germany). P2 was emulsified in equal volume of complete Freund’s adjuvant, containing 1 mg/ml *Mycobacterium tuberculosis* H37RA (Thermo Fisher Scientific). Rats were examined daily starting at the day of immunization in a blinded fashion. The determination of the disease score was based on Enders et al. 1998 [26]: 0 normal; 1 less lively; 2 impaired righting/limb tail; 3 absent righting; 4 ataxic gait, abnormal position; 5 mild paraparesis; 6 moderate paraparesis; 7 severe paraplegia; 8 tetraparesis; 9 moribund; 10 death.

#### CFSE-labelling of Schwann cells and detection in the sciatic nerve via immunohistochemistry and flow cytometry

To examine the ability of intrathecal applied SCs to migrate into the sciatic nerve, we labeled SCs with carboxyfluorescein succinimidyl ester (CFSE, Thermo Fisher Scientific) according to the manufacturer’s protocol based on experiments from Hou and colleagues [27]. Afterwards, we injected the labeled SCs intrathecally in 7–8-week-old female Lewis rats 11 days after EAN induction as described above. Therefore, the animals were anesthetized intraperitoneally with 8 mg/kg Xylazine (Xylavet, CP-Pharma, Burgdorf, Germany) and 80 mg/kg Ketamine (CP-Pharma). In comparison to a previous experiment transplanting 2,100,000 SCs in 3 μl intraventricularly in the experimental autoimmune encephalomyelitis animal model [28] and in consideration of the number of rats needed for primary SCs cultivation, we decided to transfer 1,000,000 SCs per animal in a volume of 15 μl. The intrathecal injection was performed as described recently [22, 29], slowly within 4 s by using a microsyringe with a 30-G needle into the fourth to fifth lumbar intervertebral space. The correct placement of the injection was confirmed by a movement of the tail (“tail flick”) as described by Fairbanks [30].

Four days after transplantation, the sciatic nerves were analyzed for CFSE-labeled SCs migration. After transcardial perfusion with PBS, both sciatic nerves were dissected and embedded in Neg-50 (Thermo Fisher Scientific), snap frozen, and sectioned (8 μm) on a cryostat (Thermo Fisher Scientific) for immunohistochemical proof. After mounting on deep frozen approved glass slides (Hartenstein, Würzburg, Germany), tissue was analyzed with a microscope (BX51; Olympus, Hamburg, Germany) equipped with an Olympus DP50 digital camera.

For flow cytometry, cells were isolated from the sciatic nerves based on the protocol of Liu and colleagues [31]. Therefore, both sciatic nerves were collected and placed in HBSS medium. Nerves were stripped of epineurium and sectioned into 1–2 mm pieces. Explants were dissociated for 30 min in 70 U/ml papain (Cell Systems, Troisdorf, Germany) in HBSS at 37 °C. Afterwards, 1 ml HBSS enriched by 25 mM HEPES (4-(2-hydroxyethyl)-1-piperazineethanesulfonic acid, Thermo Fisher Scientific) and 10% horse serum (Thermo Fisher Scientific) was added and nerves were suspended and filtered through a 100-μm strainer. Flow cytometry of medium control and CFSE labeled SCs transplanted EAN rats were performed as described above.

#### Nerve conduction tests

Nerve conduction tests were performed by a blinded investigator at the end of the experiment at day 20 post immunization as described earlier [21, 22]. In short, animals were anesthetized intraperitoneally with Xylazine and Ketamine. A fully digital recording Keypoint apparatus (Neurolite AG, Belp, Switzerland) was used in combination of paired needle electrodes, which were inserted into the sciatic notch (hip, proximal) and into the talocrural region (ankle joint, distal). The sciatic nerve was stimulated with supramaximal rectangular pulses of 0.05-ms duration, and the resulting compound muscle action potential was recorded from needle electrodes placed subcutaneously over the dorsal foot muscles. A ground electrode was placed between the distal stimulating electrode and the active recording electrode. To calculate the motor nerve conduction velocity, the distance between stimulating cathodes was divided by the difference of the latency. Similarly, the persistence and minimum latency of ten F-waves evoked by stimulation at the popliteal fossa were recorded for the right side [32, 33]. Temperature differences were minimized by conducting the study as soon as the anesthesia had taken effect and by warming the leg with a heating lamp.

#### Immunohistochemical staining of CD3, CD68, and fluoromyelin

Sciatic nerve extraction and histological preparation were performed as described above. For the immunohistochemical staining, cryostat sections were fixated in acetone at 20 °C for 10 min and were exposed to the mouse monoclonal antibodies (mAb) anti-rat 15-6A1 (Pan T-Cells CD3, 1:100, Hycultec, Beutelsbach, Germany) and anti-rat ED1 (anti-CD68, macrophages, 1:100, Hycultec). Secondary antibodies conjugated with Alexa 555 (1:1000) or Alexa 488 (1:1000) (Thermo Fisher Scientific) were used according to manufacturer’s protocol. DAPI (Biozol Diagnostica Vertrieb) was used for fluorescent staining of DNA. We identified the demyelination through the accumulation of nuclei and absence of FluoroMyelin™ Red fluorescent stain (1:300, Invitrogen) performed according to the manufacturer’s protocol. Omission of the primary antibodies served as negative control.

### Statistical method

Statistical analyses were performed by GraphPad Prism 7 software (GraphPad Software Inc., San Diego, CA, USA). Data are provided as mean ± SEM (standard error of mean). Differences between pairs of groups were tested by Mann-Whitney *t* test. Differences between three or more groups were tested by one-way analysis of variance (ANOVA) combined with Kruskal-Wallis test. Probability levels (*p* values) are indicated as * = *p* < 0.05, ** = *p* < 0.01, *** = *p* < 0.001, and **** = *p* < 0.0001.

## Results

### Capsaicin induces increased resistance to oxidative stress and an anti-inflammatory profile in Schwann cell monoculture

**Fig. 1:**
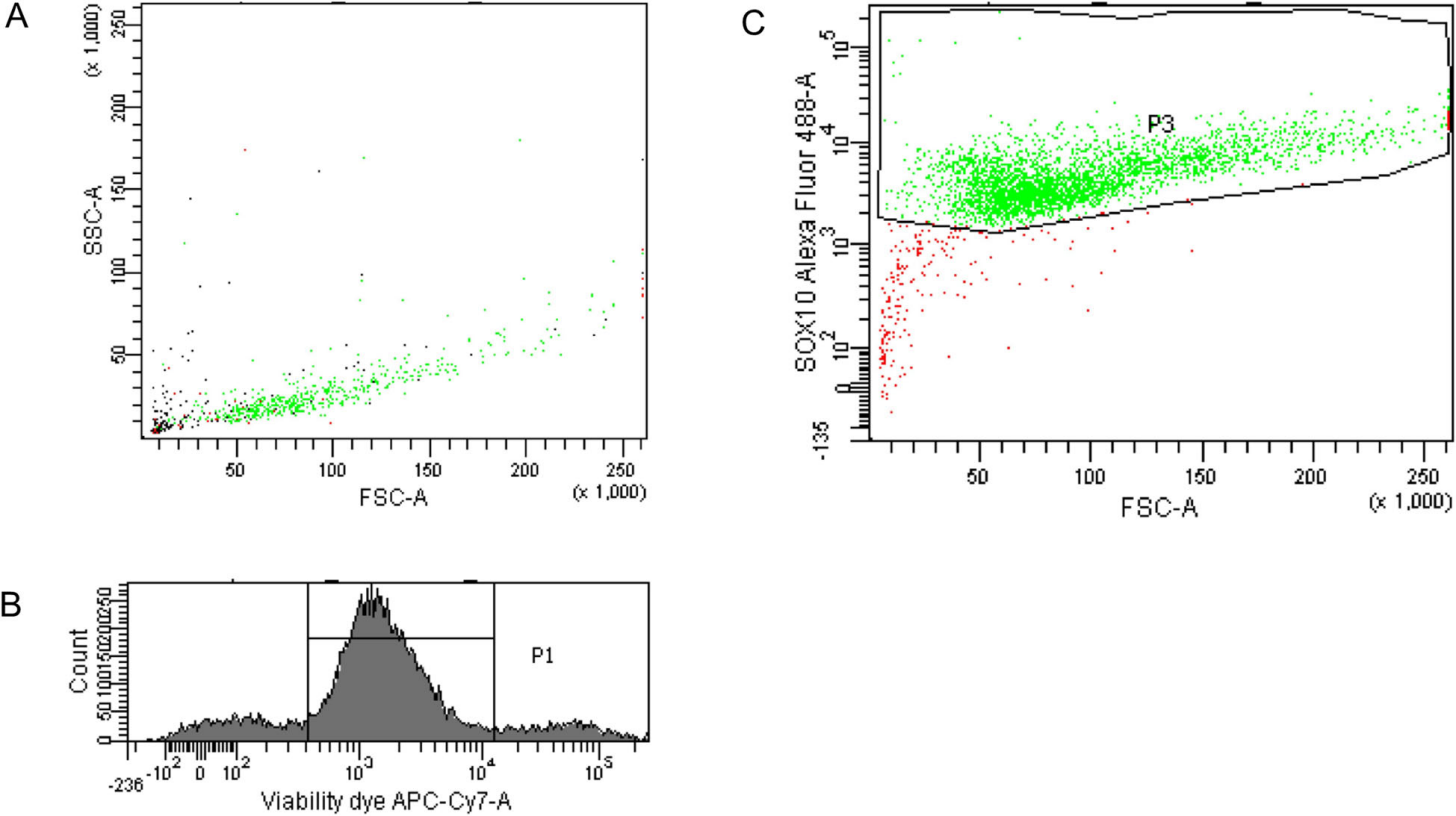
In order to analyze SC monoculture purity, we stained for SOX10 as a SC marker after selection of viable cells (fixable viability dye eFluor 780). SC monoculture showed a purity of > 90%

Analyzing immunocytochemistry (S-100 and SOX10 staining) and flow cytometry (SOX10 labeling), SC monoculture showed a purity of > 90% (Fig. 1).

**Fig. 2:**
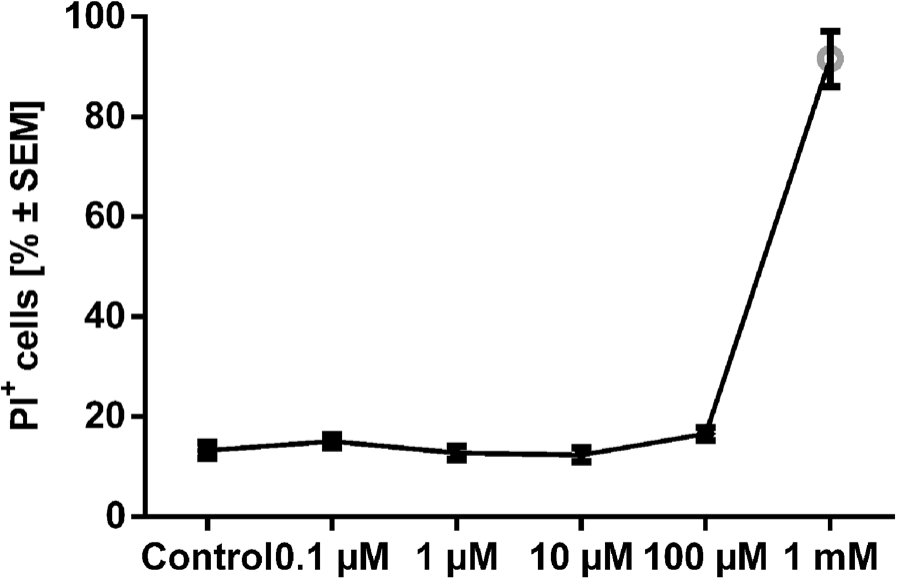
SC incubation with capsaicin over 24 h. Capsaicin exerts a toxic effect on SCs at a concentration of 1 mM in FACS analysis of PI staining. *n* = 5–16, **p ≤ 0.01

In titration experiments, there was a significant toxicity of 1 mM capsaicin dissolved in DMSO compared to a medium control in a 24-h incubation time via PI staining (Fig. 2, one-way ANOVA combined with Kruskal-Wallis test, *n* = 5–16, *p* < 0.01). There was no evidence of toxicity (PI staining) after SC incubation with 10 μM capsaicin over 16 days (data not shown).

**Fig. 3:**
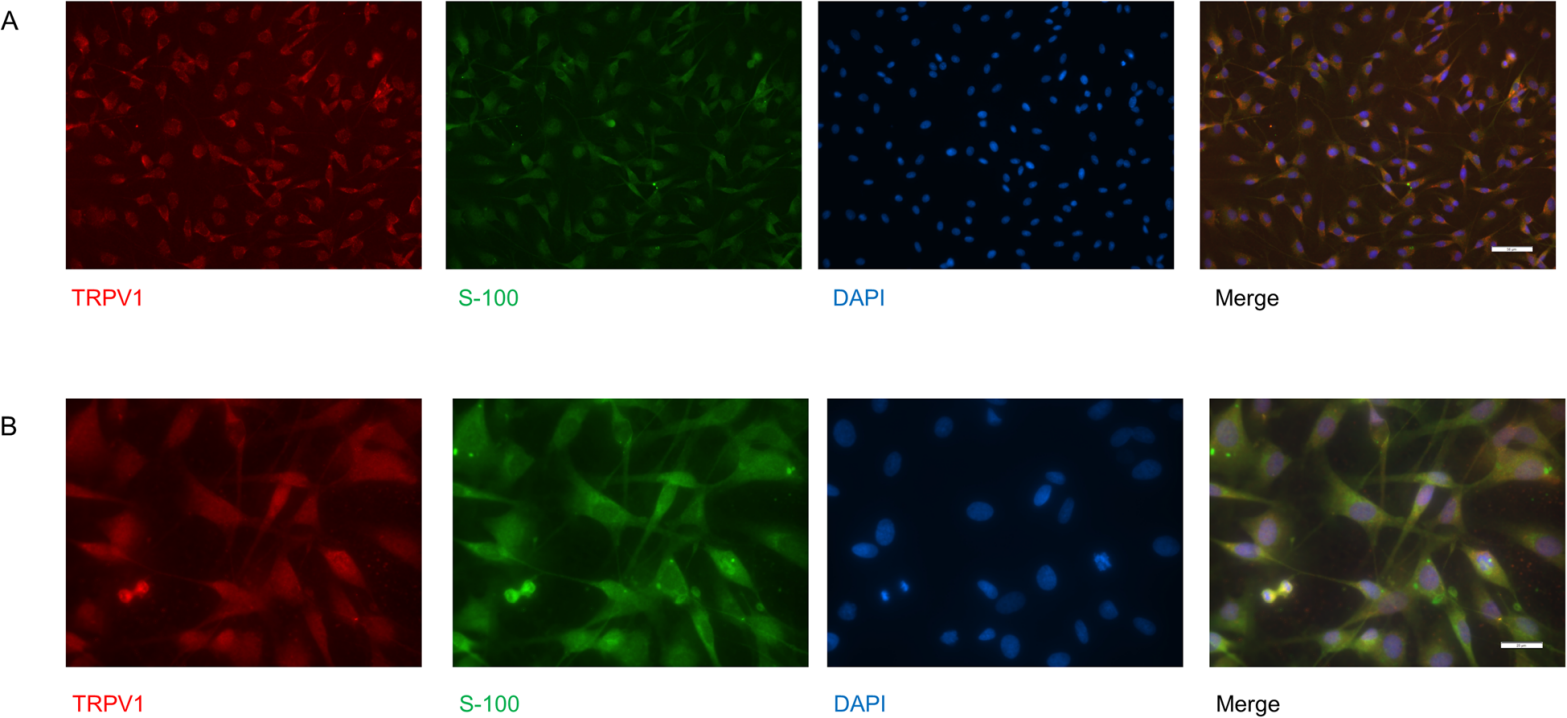
Evidence of TRPV1 on SCs by immunocytochemical staining. SC were labeled with S-100, nuclei were counterstained with DAPI. **a** × 40 magnification. Scale bar 50 μm. **b** × 100 magnification. Scale bar 20 μm

Previously, the expression of TRPV1 of SCs was suspected because of a colocalization of S-100 and TRPV1 in sciatic nerve cryo stains [21]. Here, we were able to prove TRPV1 expression on SCs via immunocytochemical labeling and in qrt-PCR in a SC monoculture. Representative pictures of SC staining with TRPV1, S-100, and DAPI are presented in Fig. 3.

**Fig. 4:**
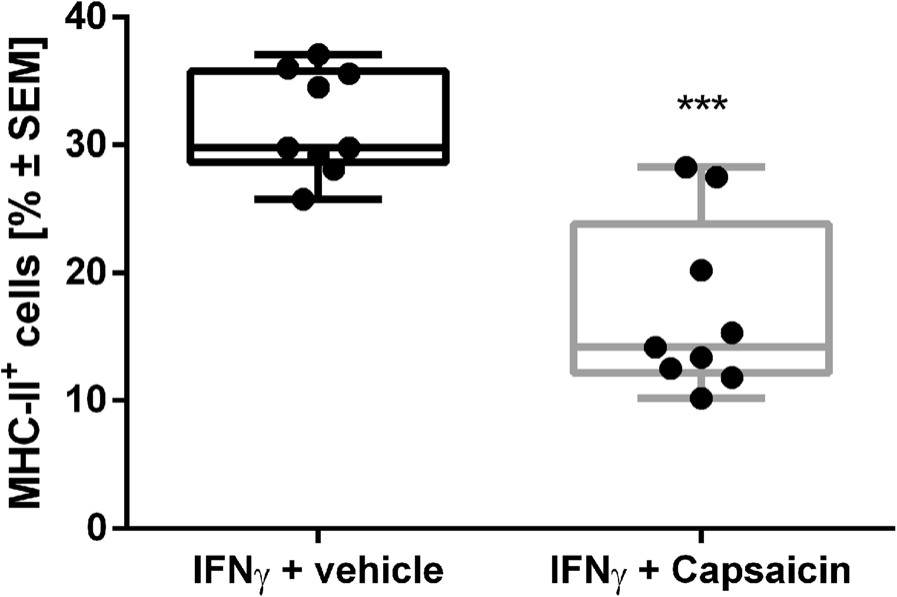
We were able to generate a submaximal MHC-II surface presentation after SC incubation with 100 U/ml IFN gamma for five days. Simultaneous capsaicin treatment (10 μM) reduced MHC-II presentation significantly. *n* = 6, ***p ≤ 0.001

In nerve biopsies of GBS and CIDP patients, but not in healthy controls, there is evidence of MHC-II presentation in SCs [34–36]. Since SCs act as facultative antigen-presenting cells, they only show MHC-II receptor surface presentation after induction in an inflammatory environment. It has been shown that SCs presented MHC-II in a sub-maximal manner after incubation with 100 U/ml IFN gamma for 5 days [22, 24]. A parallel incubation with 10 μM capsaicin significantly reduced MHC-II presentation from 47.6 to 25.6% in FACS analysis (Fig. 4, Mann-Whitney *t* test, *n* = 6, *p* < 0.001).

**Fig. 5:**
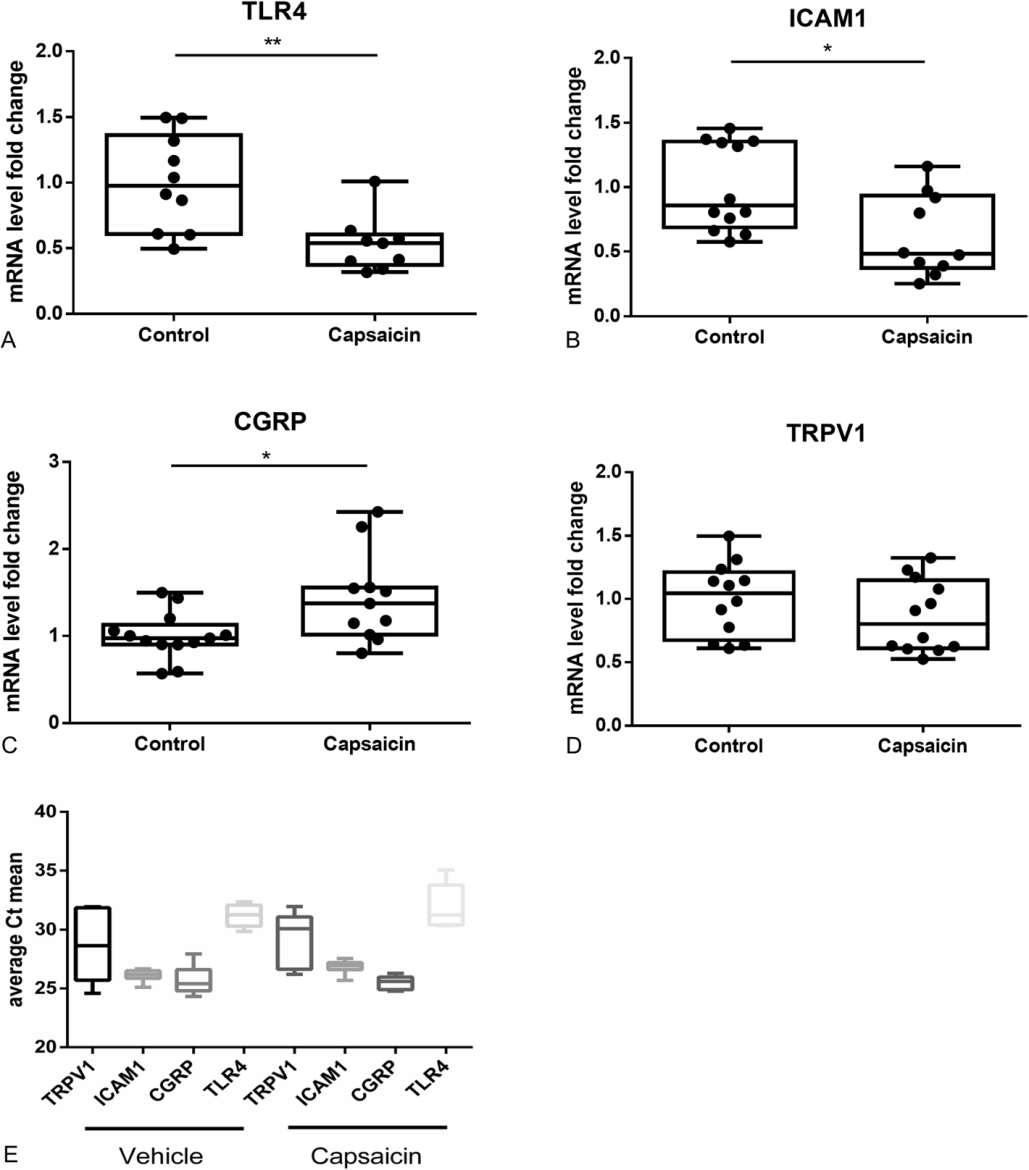
Gene expression profile after capsaicin treatment. 24-h SC incubation with 10 μM capsaicin reduces significantly TLR4 (**a**, *n* = 9–10) and ICAM-1 (**b**, *n* = 10–12) expression and induces CGRP (**c**, *n* = 11–13) expression. TRPV1 (**d**) expression is not affected. The relative quantitation of each mRNA was performed using the comparative Ct method. A comparison of the mean Ct values can be found in Fig. 4e. The target gene expression levels were normalized to the geometric average of two housekeeping genes (beta-actin, GADPH). We applied each sample in two technical replicates. *p ≤ 0.05, **p ≤ 0.01

We analyzed the ICAM-1 and TLR4 expression after capsaicin treatment, because SCs express these proteins under inflammatory conditions [37] and a pathogenic role of both in inflammatory autoimmune-mediated neuritis is suspected [38–40]. After 24 h of capsaicin (10 μM) treatment, we were able to detect a significant reduction in the expression of TLR4 (Fig. 5a, Mann-Whitney *t* test, *n* = 9–10, *p* < 0.01) and ICAM-1 (Fig. 5b, Mann-Whitney *t* test, *n* = 10–12, *p* < 0.05) compared to a medium control in qrt-PCR. Capsaicin did not induce TNF alpha, IL-4, IL-10, and IFN gamma expression.

An effect of capsaicin on both, TRPV1 and its downstream molecule CGRP, has been demonstrated in previous in vivo studies [21]. Both proteins have a key function in the neuro-immune axis [41]. We were able to illustrate a significant increase of CGRP (Fig. 5c, Mann-Whitney *t* test, *n* = 11–13, *p* < 0.05), but not of TRPV1 expression (Fig. 5d) in qrt-PCR.

**Fig. 6:**
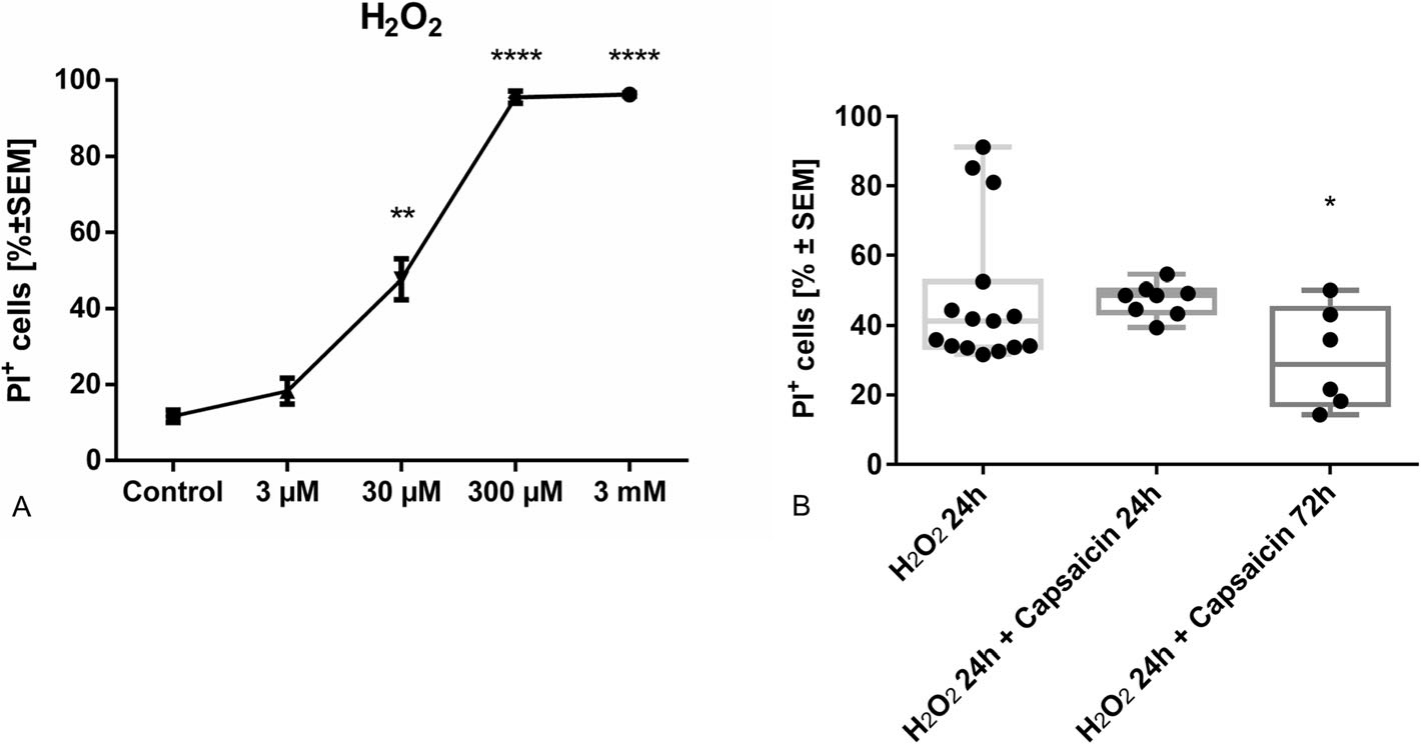
**a** FACS analysis of cell toxicity of different H_2_O_2_ concentrations (3 μM to 3 mM; incubation over 24 h) on SC. A sub-lethal condition was conducted at 30 μM. **b** Incubation of SCs for 24 h with 30 μM H_2_O_2_ with simultaneous treatment of capsaicin or vehicle to examine the protective role of capsaicin in the context of oxidative stress. In order to simulate a protective effect of capsaicin at a preventive setting, these experiments were repeated with further pre-treatment with 10 μM capsaicin or vehicle for 48 h. Pre-treatment with capsaicin was required to significantly reduce lethality induced by 30 μM H_2_O_2_. *n* = 6–-15, *p ≤ 0.05, **p ≤ 0.01, ***p ≤ 0.001, and ****p ≤ 0.0001

Incubation of SCs with 30 μM H_2_O_2_ over 24 h generated a sub-maximal lethal condition (47.7%) detected in PI-staining with flow cytometry (Fig. 6a). After having found a sublethal H_2_O_2_ concentration, we tried to demonstrate a potential anti-oxidative effect capsaicin. However, simultaneous incubation of SC with H_2_O_2_ and capsaicin or vehicle did not show any difference in cell death rates. Only pre-treatment with 10 μM capsaicin for 48 h reduced cell death significantly to 30.5% (Fig. 6b, one-way ANOVA combined with Kruskal-Wallis statistic, *n* = 6–15, *p* < 0.05).

### Capsaicin does not influence myelination in vitro

**Fig. 7:**
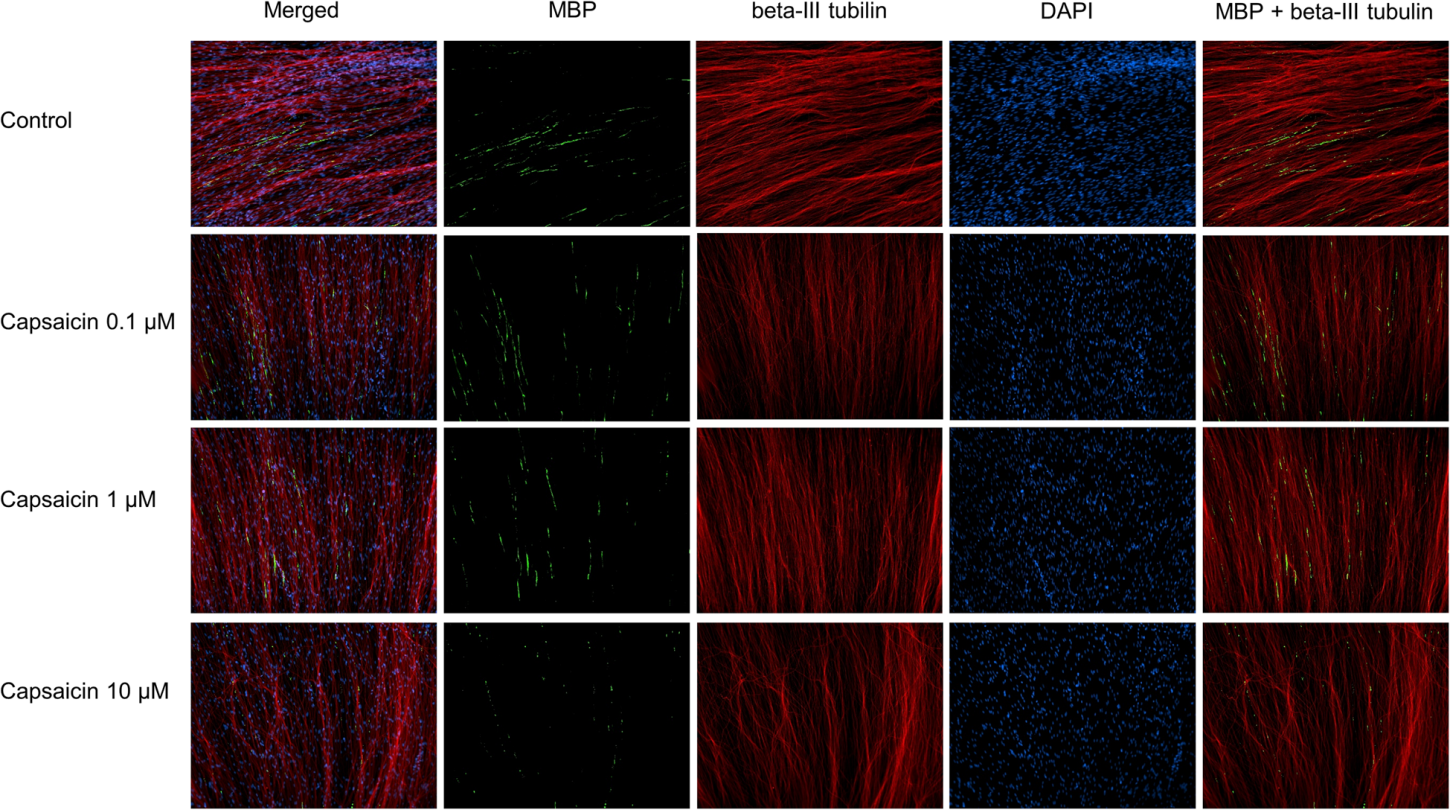
Comparison of the rate of myelinated axons between naïve and capsaicin treated (0.1 μM, 1 μM, and 10 μM) SC-DRG coculture at day 14. Myelination were verified by MBP staining. Axons and nuclei were stained by beta-III tubulin and DAPI, respectively. × 20 magnification. Scale bar 200 μm

**Fig. 8:**
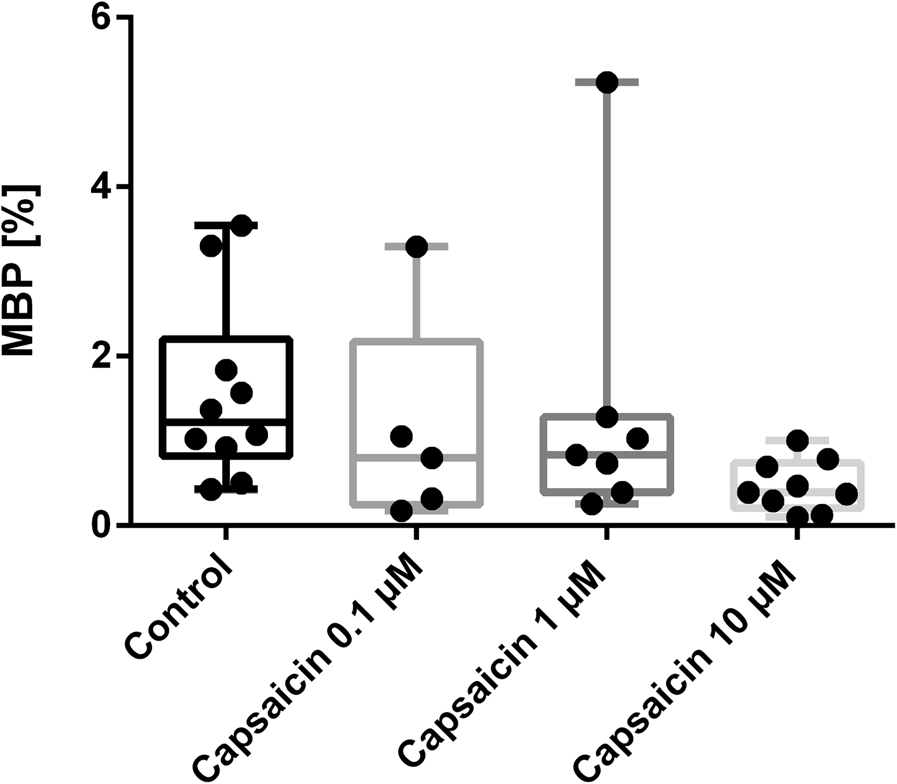
Coculture treatment with 0.1 μM, 1 μM, or 10 μM capsaicin was not able to influence myelination in vitro, *N* = 3–5

We were able to implement a rapid myelination model in vitro by coculture SCs from adult rats and DRG from prenatal day 14 (Fig. 7). Myelination verified by MBP staining was visible at day 10 and increased in the following 6 days. Simultaneous treatment with 0.1 μM, 1 μM, or 10 μM capsaicin did not influence MBP staining at day 14 after applying SCs in the DRG-culture (Fig. 8, one-way ANOVA combined with Kruskal-Wallis statistic, *N* = 3–5). Representative pictures of immunocytochemical staining (beta-III-tubulin, MBP, and DAPI) of the naïve SC-DRG-coculture and after capsaicin treatment are presented in Fig. 7.

### Intrathecally transplanted Schwann cells ameliorate EAN pathology with no further beneficial effect of capsaicin pre-treatment

**Fig. 9:**
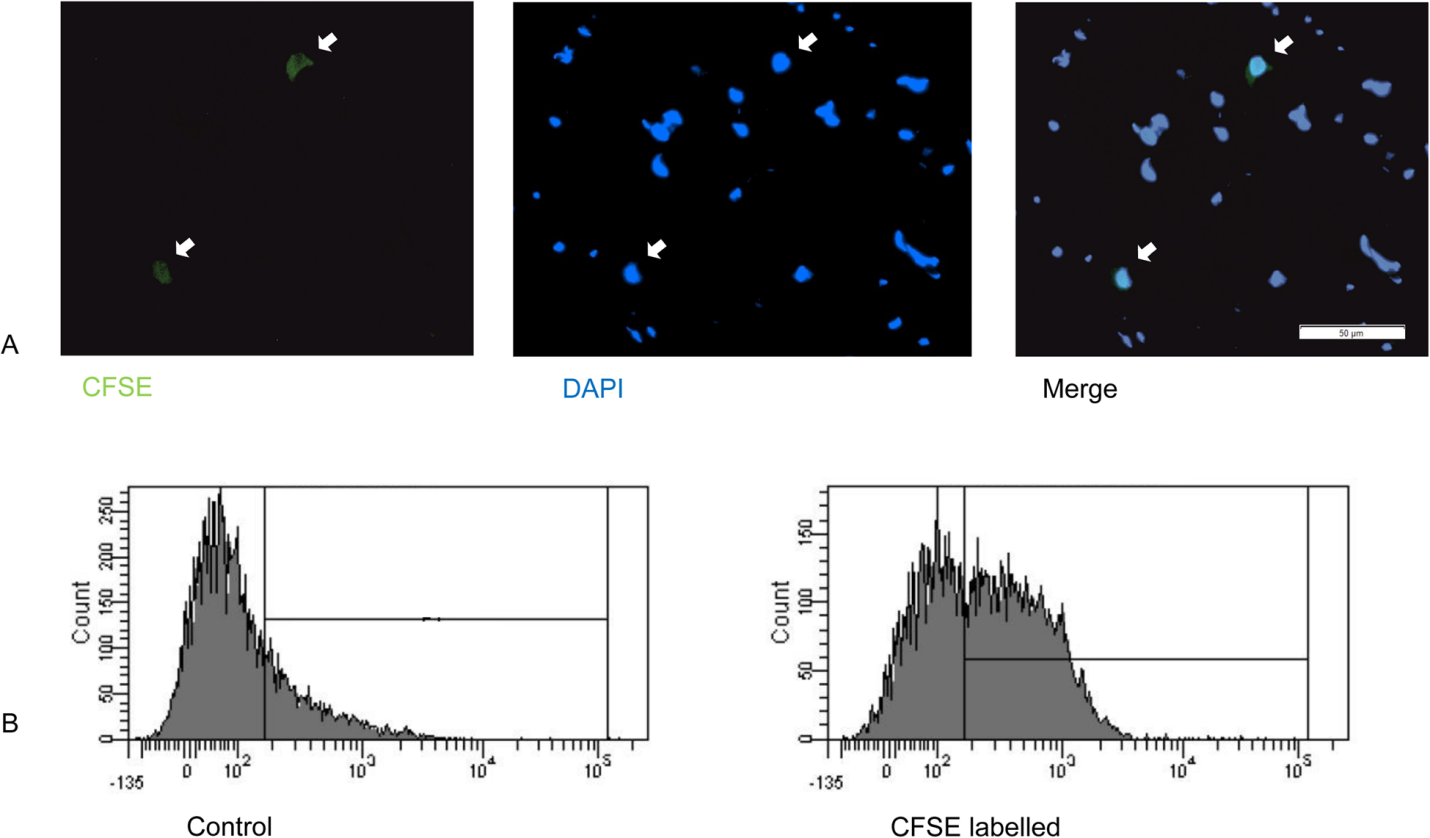
SCs were labeled with CFSE and intrathecally transferred in EAN rats at day 11. Four days later, these CFSE labeled SCs can be detected in histologically (**a**) in cryo sections of the sciatic nerve and via flow cytometry (**b**) after digestion of the sciatic nerve. × 40 magnification. Scale bar 50 μm

After transplantation of CFSE-labeled SCs in the spinal fluid of EAN rats, we were able to detect a CFSE signal histologically (Fig. 9a) and in flow cytometry (Fig. 9b) after 4 days in the sciatic nerves.

**Fig. 10:**
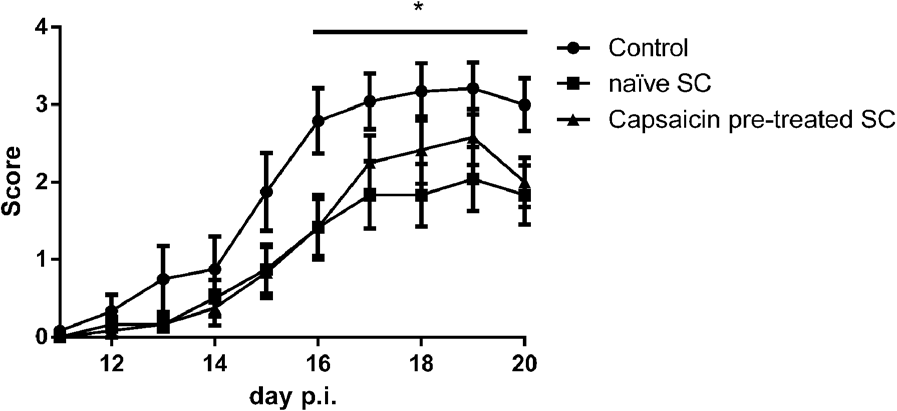
Transfer of naïve, but not capsaicin pre-treated SCs intrathecally in EAN rats significantly ameliorated clinical symptoms in the EAN rats. *n* = 12, *p ≤ 0.05

Having transplanted naïve SCs intrathecally, clinical symptoms in the EAN rats significantly ameliorated (*n* = 12, multiple *t* test, d16–20: *p* < 0.05). Capsaicin pre-treated SC had no significant beneficial effect on the EAN course (Fig. 10).

**Fig. 11:**
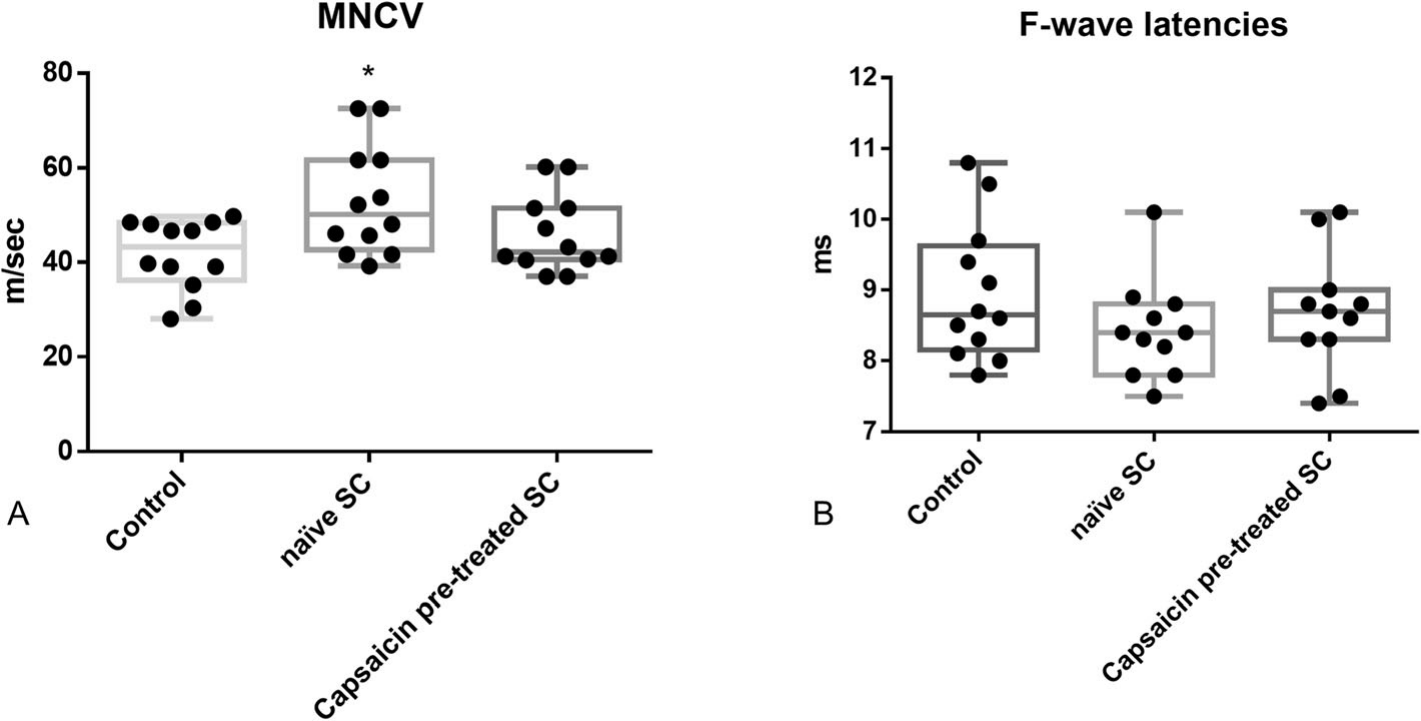
After intrathecal transfer of naïve SCs in EAN rats, motor nerve conduction velocity (**a**), but not F-wave latencies (**b**), were significantly ameliorated at day 20 after immunization. Capsaicin pre-treatment did not significantly influence nerve conduction. *n* = 12, *p ≤ 0.05

Electrophysiological evaluation of the sciatic nerve at day 20 after immunization revealed significant increased motor nerve conduction velocity in the SC-treated group in comparison to the PBS-treated group (Fig. 11a, one-way ANOVA combined with Kruskal-Wallis statistic, *n* = 12, *p* < 0.05). No significant effect was measured regarding F-wave latency (Fig. 11b).

**Fig. 12:**
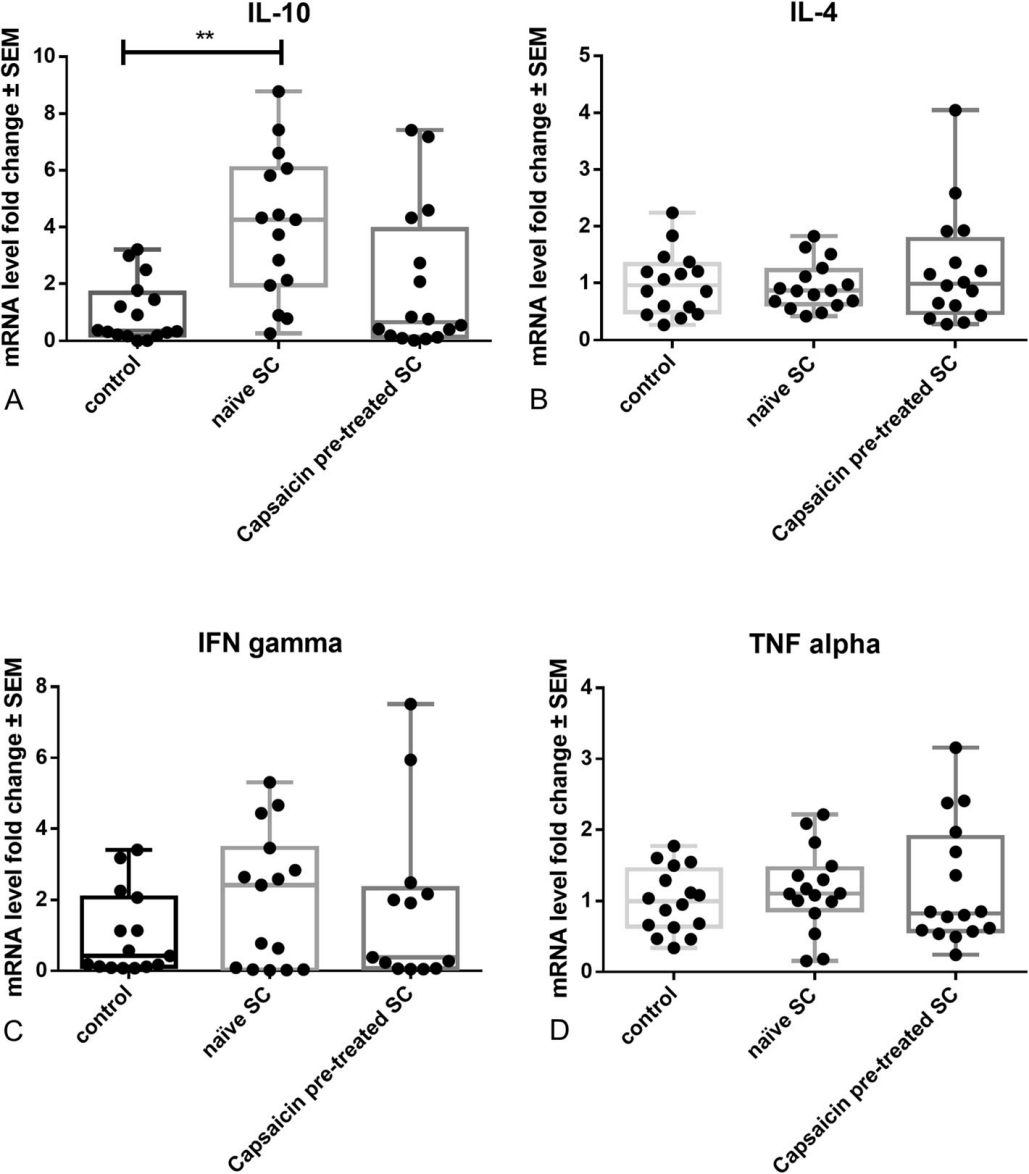
Intrathecal transfer of naïve, but not capsaicin pre-treated SCs in EAN rats significantly induces IL-10, but not IL-4, IFN gamma, or TNF alpha expression in the sciatic nerve. *n* = 12, **p ≤ 0.01

We performed qrt-PCR analysis. Here, we were able to demonstrate a significant upregulation in IL-10 (one-way ANOVA combined with Kruskal-Wallis statistic, *n* = 12, *p* < 0.01), but not IL-4, IFN gamma, or TNF alpha expression in naïve SCs transplanted EAN rats (Fig. 12a–d).

**Fig. 13:**
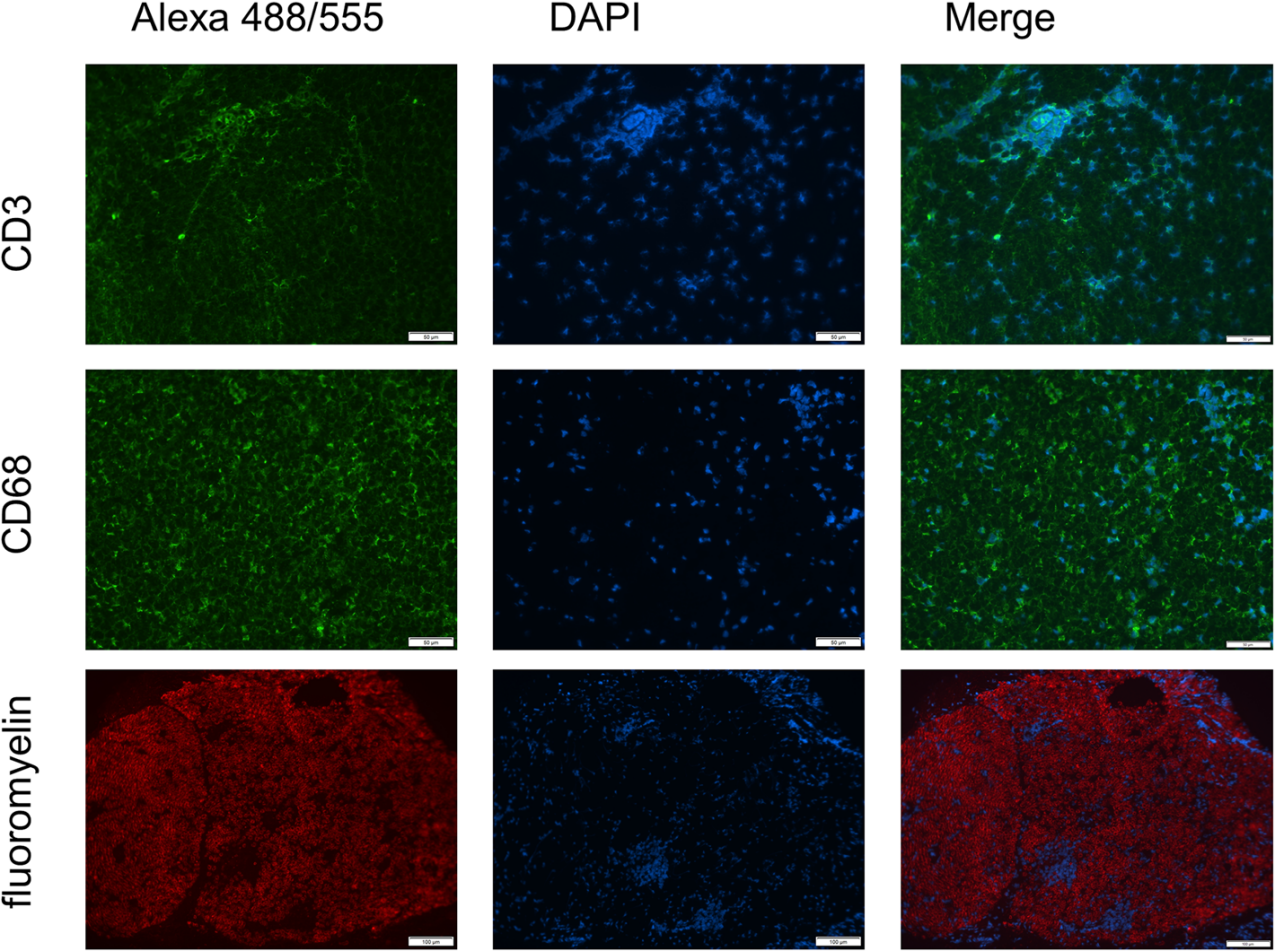
Representative figure of immune cell infiltration in CD3 and CD68 staining (× 40 magnification, scale bar 50 μm) and a demyelinating lesion in fluoromyelin staining (× 10 magnification, scale bar 100 μm) in EAN pathology

Inflammation and demyelination in the EAN rats have been proved by CD3, CD68, and fluoromyelin staining on a random basis (Fig. 13).

## Discussion

In our current study, we elucidated an immunomodulatory and anti-oxidative role of capsaicin on SCs. These results are in line with our previous work and provide another putative explanation for the preventive effect of capsaicin in immune-mediated neuropathies. Based on our previous study, an anti-inflammatory effect of oral capsaicin, that was admitted before immunization in EAN rats in a preventive setting, was already known. Using capsaicin as a therapeutic treatment (starting treatment at the day of immunization) did not reduce clinical and paraclinical sign of neuritis [21].

In the present work, a reduced IFN gamma-induced MHC-II presentation was evident after capsaicin treatment in vitro. This provides evidence for the anti-inflammatory role of SC in the amelioration of EAN pathology after capsaicin consumption. Since SCs act as facultative antigen-presenting cells, they only show MHC-II receptor presentation after induction in an inflammatory environment. Likewise, in nerve biopsy of GBS and CIDP patients, but not in healthy controls, there is evidence of MHC-II presentation of SCs [34–36] as well as upregulation of the co-stimulatory receptor BB-1 and an interaction with endoneurial T cells [42]. The functional relevance of SCs for effector T cells has been confirmed in cell culture experiments: In the presence of ovalbumin peptide, Schwann cells induce the proliferation of ovalbumin-specific CD4^+^ T cells, whereas the presence of ovalbumin alone is not sufficient to induce T cell proliferation [43]. Comparable results have been shown with different antigens [44]. Furthermore, reduced post-traumatic intraneural inflammation and neuropathic pain were evident in the chronic constriction injury mouse model after conditionally knocking out MHC-II β-chain from myelinating SCs [45]. In summary, there is striking evidence that SCs participate in the inflammatory process in autoimmune-mediated neuritis and that reduced MHC-II presentation has a regulatory effect.

We demonstrated a significant reduction of ICAM-1 and TLR4 expression in SCs after capsaicin treatment. ICAM-1 is a membrane glycoprotein initiating migration of leukocytes during inflammation. Similar to MHC-II synthesis, SCs express ICAM-1 under inflammatory conditions, for instance by IFN gamma stimulation [37]. A pathogenic role of ICAM-1 in inflammatory autoimmune-mediated neuritis is suspected as increased ICAM-1 expression in the serum of GBS and CIDP patients was seen [38]. In line with this, Yosef and Ubogu [46] showed an increased leukocyte migration through the human blood-nerve barrier due to upregulated ICAM-1 expression on primary endoneurial endothelial cells. Since SCs provide similar claudin-containing junction like endothelial cells of the blood-nerve barrier [47], ICAM-1 reduction after capsaicin treatment might have a direct anti-inflammatory effect through lymphocyte migration reduction.

TLR4 is part of the innate and adaptive immune responses and can be induced by lipopolysaccharides and other pathogen- and damage-associated molecular patterns. Thereby, TLR4 increases macrophage stimulation and antigen presentation. In the serum of GBS patients [39, 40] and at the peak of disease course in spleen, lymph node, peripheral blood, and sciatic nerve in the EAN animal model, TLR4 mRNA expression is increased [48]. Thus, a downregulation through capsaicin treatment might participate in reduction of local nerval inflammation.

Furthermore, we showed that capsaicin increased the resistance of SCs against oxidative stress only after pre-treatment. In human patients, the presence of free radicals is the main parameter contributing to inflammation and subsequent neurodegeneration, but myelin is also highly vulnerable to oxidative stress due to its high lipid content. Measurements of the highly reactive malondialdehyde concentration in cerebral spinal fluid in GBS patients indicated increased oxidative activity in this disease that might cause membrane damage and subsequently SC and neuronal apoptosis [49–51]. Damaged SCs may perpetuate epitope spreading, which maintains an immune response. Different SC-epitopes are known as targets for inflammatory autoimmune-mediated neuritis [52]. Interestingly, malondialdehyde concentrations were reduced in GBS patients after immunotherapy [50]. Both emphasize the striking role of oxidative stress in inflammatory autoimmune-mediated neuritis and, thereby, reveal a potent beneficial approach for reducing oxidative stress. Similarly, our group has shown that triamcinolone, a corticosteroid used intrathecally for CIDP treatment, increased SC resistance to oxidative stress [22].

Interestingly, we detected an upregulation of CGRP expression. TRPV1 expression did not change. Capsaicin is a direct agonist of TRPV1, and CGRP is a downstream molecule of TRPV1. Formerly, it was believed that neuropeptides, such as CGRP, are exclusively synthetized by neurons, but its expression in non-neuronal cells such as monocytes was recently demonstrated [53, 54]. CGRP acts as a key neurotransmitter in the neuro-immune axis: CGRP is able to promote Th2 immunity, regulate MHCII expression, and promote anti-inflammatory cytokines and receptor expression [41]. Furthermore, CGRP reduced oxidative stress in SCs after lentivirus-mediated overexpression of CGRP [55]. In former studies, it has been shown that CGRP can inhibit lipopolysaccharide-induced TNF-alpha synthesis in peripheral nerves [56]. In our previous in vivo experiments of orally given capsaicin in the EAN animal model, a trend (*p* = 0.0681) to an increasing CGRP expression and significant upregulation of TRPV1 in the sciatic nerve was evident [21]. Nevertheless, in analyses of the complete sciatic nerve, different cell types could not be distinguished. Our results indicated, at least for CGRP expression, that the increase of CGRP expression in SCs is relevant in the capsaicin-mediated anti-oxidative and modulatory effect. However, further experiments must clarify if other cell types than SC in the sciatic nerve or infiltrating cells promote TRPV1 increasing as a reaction to capsaicin treatment, particularly in the context that in our in vivo experiments an upregulation of TRPV1 was also demonstrated in the small intestine [21].

Since SCs have the dual function of immunomodulation and myelination, we decided to additionally investigate the effect of capsaicin on myelination in a SC-dorsal root ganglion-coculture. We were able to implement a coculture of SC and axons from DRG and provided evidence of myelination in vitro. Capsaicin did not modify myelination in this stable coculture system. Since we have illustrated an anti-oxidative and immunomodulatory effect of capsaicin in vitro and, moreover, in the EAN model in vivo [21], further studies are warranted to demonstrate if capsaicin diminishes demyelination and inflammation in a coculture under inflammatory condition and/or oxidative stress. Up to now, there seems to be no effect on myelination in non-inflammatory conditions. These results are similar to the effect of fingolimod on SCs and the EAN animal model. Köhne and colleagues [57] demonstrated that fingolimod, approved for multiple sclerosis in 2010, did not support myelination; however, strong evidence exists for an attenuating effect of fingolimod in EAN due to immune modulation and axonal protection [58]. Finally, it was shown that SCs transit to a phenotype that promotes nerve regeneration without increasing myelination after fingolimod treatment [59, 60]. Further studies are necessary to investigate the positive impact of capsaicin in EAN.

In order to confirm the immunomodulatory effect of capsaicin on SC and its relevance in the in vivo model, we transferred naïve and capsaicin-treated SCs intrathecally at the peak of disease in EAN rats. For the first time, we have shown that naïve intrathecal transplanted SCs ameliorate symptoms, improve nerve conduction velocity in the EAN, and promote an upregulation of IL-10 expression. IL-10 exerts a complex anti-inflammatory role through expression by almost all cells of the innate and adaptive immune system, including Th1, Th2, Th17, regulatory T cells, regulatory (M2) macrophages, and SCs [61]. There is evidence that Treg cells respond via IL10 receptors to IL-10 production of other immune effector cells and boost regulatory mechanisms [62]. In macrophages, IL-10 leads to IL-10 receptor autophosphorylation, activation of the transcription factor STAT3, and subsequent inhibition of pro-inflammatory cytokine expression [63]. Therefore, an IL-10 expression after naïve SC transfer might amplify an anti-inflammatory cascade. A role of IL-10 in immune neuropathies has been indicated after administration of IL10 in EAN rats reducing their severity and has been associated with a Th1-to-Th2-shift [64].

On the other side, the expression of IL-10 of SCs is increased during Wallerian degeneration [65]. Besides the anti-inflammatory effect, IL-10 provides direct trophic support to neurons due to a neuronal IL-10-receptor [66]. In a study of lateral hemisection injury, artificially upregulated IL-10 expression resulted in increased neuronal survival and improved motor function [67]. Moreover, it has been shown that administration of IL-10 to a side of sciatic nerve injury improved regeneration [68].

A previous study was only able to demonstrate a positive effect of SCs that were transplanted in the cisterna magna in EAN rats after pre-treatment with brain-derived neurotrophic factor, even though in this study noticeable fewer amount of SCs were transplanted [27]. Furthermore, intrathecally applied SCs improved inflammation after spinal cord injury [69] and in the experimental autoimmune encephalomyelopathy, the animal model of multiple sclerosis [28]. These results have led to a phase I-trial that examines the safety of autologous transplanted SCs after subacute thoracic spinal cord injury [70]. In the light of these studies, our results might be beneficial for potent therapy options in CIDP.

Capsaicin-treated SCs did not show any further beneficial effect on clinical signs in EAN. Recent studies have shown that mature SC must re-differentiate to “repair SC” to provide remyelination. In mature SCs, a complex cascade of transcription factors that drive protein and lipid expression and allow remyelination is actively suppressed and has to be activated mainly driven by an upregulation of SOX-10 [71, 72]. As capsaicin-ameliorated clinical signs in the EAN animal model [21], these results might indicate an anti-oxidative und immunomodulatory effect by capsaicin but no direct influence on myelination or re-differentiation. Furthermore, we cannot rule out that capsaicin might have a primary effect on non-myelinating SCs. On the other side, it might be a sufficient approach to transfer capsaicin pre-treated SCs in a preventive setting at the day of immunization, which was not included in our current studies. Furthermore, the duration of capsaicin treatment in SCs might have been too short to sustainably modulate the SCs. The exact differentiation type of SCs under capsaicin treatment should be focused in further studies.

Interestingly, our model enlightens further TRPV1 and CGRP-mediated processes being pathologically essential in the context of polyneuropathies. TRPV1 and its downstream molecules CGRP and Substance P are important for detection of nociception and thermal inflammatory pain as revealed by experiments on knock-out animals [73–75]. Recent studies have shown that an increased expression of TRPV1 in peripheral nerve terminals mediate thermal nociception in the Fabry disease mouse model [76]. Furthermore, TRP channels and CGRP are involved in migraine pathology and a first CGRP antibody, Erenumab, has been approved by the EMA for migraine treatment [77]. Therefore, further studies could reveal a systemic role of capsaicin in modulation of nociception.

## Conclusion

To conclude, we have shown that capsaicin exerts an immunomodulatory and anti-oxidative effect on SCs. These results are in concordance with an anti-inflammatory effect of orally given capsaicin in EAN in a preventive setting [21]. In the present work, we have shown for the first time that SCs increase CGRP expression after capsaicin treatment and might have a key role in the anti-inflammatory effect of capsaicin in autoimmune neuritis. Aspects of the anti-inflammatory function of capsaicin in SC are reduced MHC II presentation after INFy stimulation, and downregulated TLR4 and ICAM-1 expression. Furthermore, we were able to demonstrate an increased anti-oxidative potential. Since we were not able to demonstrate a beneficial effect of capsaicin on myelination in a naïve SC-SRG coculture system and of capsaicin-treated SC in EAN rats, the immunomodulatory potential probably overweighs the neuroprotective/repairing potential. These results pose novel questions in the investigation of immunomodulatory nutrients for immune-mediated neuropathies as their effects seem to be sufficient if given early in the disease course. Further studies must reveal if changes in nutrition in favor of spicy food are able to exert a preventive effect for human CIDP. To clarify the entire effect of capsaicin on inflammatory autoimmune-mediated neuritis, further studies must examine alternative cell types that are involved in inflammation and the role of TRPV1 in these pathologies.

## Data Availability

The datasets used and/or analyzed during the current study are available from the corresponding author on reasonable request.

## Abbreviations

TRPV1: Transient receptor potential channel vanilloid subfamily member 1
CIDP: Chronic inflammatory demyelinating polyneuropathy
PNS: Peripheral nervous system
SC: Schwann cell
EAN: Experimental autoimmune neuritis
CGRP: Calcitonin gene-related peptide
TLR4: Toll-like receptor 4
ICAM-1: Intercellular adhesion molecule 1
DRG: Dorsal root ganglia
GBS: Guillain-Barré syndrome
DMSO: Dimethyl sulfoxide
PI: Propidium iodide
PBS: Phosphate buffered solution
DAPI: 4′,6′-Diamidino-2-phenylindole·2HCl
MHC-II: Major histocompatibility complex class II
IFN: Interferon
RNA: Ribonucleic acid
qrt-PCR: Qualitative real-time polymerase chain reaction
IL: Interleukin
TNF: Tumor necroses factor
MBP: Myelin basic protein
CFSE: Carboxyfluorescein succinimidyl ester
CMAP: Compound muscle action potential
SEM: Standard error of mean
ANOVA: Analysis of variance
*P* value: Probability level
